# Supplementary material for: Imaging biological tissue with high-throughput single-pixel compressive holography
Source: Nat Commun. 2021 Aug 5;12:4712. doi: 10.1038/s41467-021-24990-0 (PMC8342474; doi:10.1038/s41467-021-24990-0)
Supplement: Supplementary file 1 — Supplementary Information [file 41467_2021_24990_MOESM1_ESM.pdf]

Supplementary Information for "Imaging biological tissue with high-throughput single-pixel compressive holography"

Daixuan Wu,[1,2] Jiawei Luo, [1,2] Guoqiang Huang, [1,2] Yuanhua Feng, [3] Xiaohua Feng,[4] Runsen Zhang,[1,2,5] Yuecheng Shen, [1,2,*] and Zhaohui Li[1,2,6,*]

[1]Key Laboratory of Optoelectronic Materials and Technologies, School of Electronics and Information Technology, Sun Yat-sen University, Guangzhou, China, 510275

[2]Guangdong Provincial Key Labratory of Optoelectronic Information Processing Chips and Systems, School of Electronics and Information Technology, Sun Yat-sen University, Guangzhou, China, 510275

[3]Department of Electronic Engineering, College of Information Science and Technology, Jinan University, Guangzhou, China, 510632

[4]Department of Bioengineering, University of California, Los Angeles, USA, 90095

[5]Institute of Photonics Technology, Jinan University, Guangzhou, China, 511443

[6]Southern Marine Science and Engineering Guangdong Laboratory (Zhuhai), Zhuhai, China, 519000

Corresponding authors: *shenyuecheng@mail.sysu.edu.cn* and *lzhh88@mail.sysu.edu.cn*

Supplementary Note 1. Heterodyne holography

The developed high-throughput single-pixel holography (SPH) in this work relies on employing heterodyne holography to retrieve the summed optical fields on the surface of the single-pixel detector. In our system, the 532-nm laser (frequency f_0) was divided into a signal beam and a reference beam to realize Mach-Zander interferometry. Each beam was frequency-shifted after passing through an acousto-optical device. Hereby, we can presume an upshifting of f_1 in the signal beam and f_2 in the reference beam, respectively. In this circumstance, the total irradiance measured by the detector $I_n(t)$ is a time-varying signal. When the *n*-th Hadamard-like pattern $\widetilde{H}_n(\vec{r})$ is displayed,

$$\begin{aligned} I_n(t) &= \int_{S_d}^{1} \left|a\widetilde{H}_n(\vec{r})O(\vec{r})e^{-i2\pi(f_0+f_1)t} + be^{-i2\pi(f_0+f_2)t}\right|^2 \Delta t dS \\ &= \int_{S_d}^{1} \left[a^2\widetilde{H}_n(\vec{r})^2|O^2(\vec{r})| + b^2 + \mathrm{Re}\left[2ab\widetilde{H}_n(\vec{r})O(\vec{r})e^{-i2\pi(f_2-f_1)t}\right]\right] \Delta t dS \end{aligned} \tag{S1}$$

Here, $O(\vec{r})$ is the sample to be imaged. a and b denote the intensity of the signal beam and the reference beam, respectively. Δt equals the reciprocal of the bandwidth of the detector that should be much higher than the beating frequency $\Delta f = f_2 - f_1$. Based upon the physical implementation, spatial integration is performed over the active area of the detector S_d. Nonetheless, if the detector measures all the transmitted light, the law of energy conservation indicates that spatial integration can be performed on any plane of the optical path. For mathematical convenience, the conjugate plane of the sample surface S_c is chosen. By defining

$$\mathrm{DC} \equiv \int_{S_c}^{1} \left[a^2\widetilde{H}_n(\vec{r})^2|O^2(\vec{r})| + b^2\right] \Delta t dS \tag{S2}$$

$$\mathrm{AC} \equiv 2\sqrt{N} ab\Delta t \tag{S3}$$

and using the following relation

$$\frac{1}{\sqrt{N}} \int_{S_c}^{1} \widetilde{H}_n(\vec{r})O(\vec{r})dS = \tilde{a}_n e^{i\tilde{\varphi}_n} \tag{S4}$$

we get

$$I_n(t) = \mathrm{DC} + \mathrm{AC} \times \tilde{a}_n \cos(\tilde{\varphi}_n + 2\pi\Delta f t) \tag{S5}$$

In cases where light intensity in the reference beam is much stronger than that in the signal beam, AC is much smaller than DC. As a result, we generally observe a small sinusoidal signal sitting on top of a flat background. There are two major advantages of using heterodyne holography. First, the intensity of the reference beam appears in the interference term, boosting the useful signal through heterodyne gain. This gain is particularly helpful when the photons contributed from the signal beam are weak and comparable to the electronic noise of the detector. Second, the hologram evolves in time, leading to phase stepping naturally without taking additional efforts. By applying Fourier transformation to the measured $I_n(t)$ and locating the peak at Δf, one can estimate the values of $\tilde{a}_n$ and $\tilde{\varphi}_n$. Then, recalling the relationship between the Hadamard basis and the Hadamard-like pattern is $H_n(\vec{r}) = 2\widetilde{H}_n(\vec{r}) - \widetilde{H}_1(\vec{r})$, the

coefficient a_n and φ_n can be extracted as

$$a_n e^{i\varphi_n} = \frac{1}{\sqrt{N}}\int_{S_c}^{1} H_n(\vec{r})O(\vec{r})dS = \frac{1}{\sqrt{N}}\int_{S_c}^{1}\left(2\widetilde{H}_n(\vec{r}) - \widetilde{H}_1(\vec{r})\right)O(\vec{r})dS \\ = 2\tilde{a}_n e^{i\tilde{\varphi}_n} - \tilde{a}_1 e^{i\tilde{\varphi}_1} \tag{S6}$$

We note that the second term in Eq. (S6) is simply a constant value. Keeping this term during imaging reconstruction only imposes a constant background to the reconstructed images, which is acceptable for visualization. In practice, this term can be used to correct phase drifting that occurred during the data acquisition process. Specifically, in this work, the 1st order of Hadamard-like pattern was inserted as the tracking base after displaying every 16 orders of Hadamard-like patterns. The measurement of this recurring pattern was used to update the second term in Eq. (S6) over time.

Supplementary Note 2. The procedure of removing phase contaminations induced from the optical system

When reconstructing complex-valued images of a given sample, the uneven phase distribution originated from the imperfection of the optical system always contaminates the exact phase distributions of the sample. Amplitude contamination also exists but is found to be negligible in our case. These imperfections include but are not limited to the imperfect illuminating source, optical aberrations inherent in the microscopic system, and the curvature of the surface of the digital micromirror device (DMD). Fortunately, the phase contamination resulted from these imperfections is independent of the contributions from the sample. Therefore, a calibration process that directly dividing the reconstructed complex-valued field when the sample is absent, i.e., subtracting the phase distribution, can be used to correct these phase contaminations.

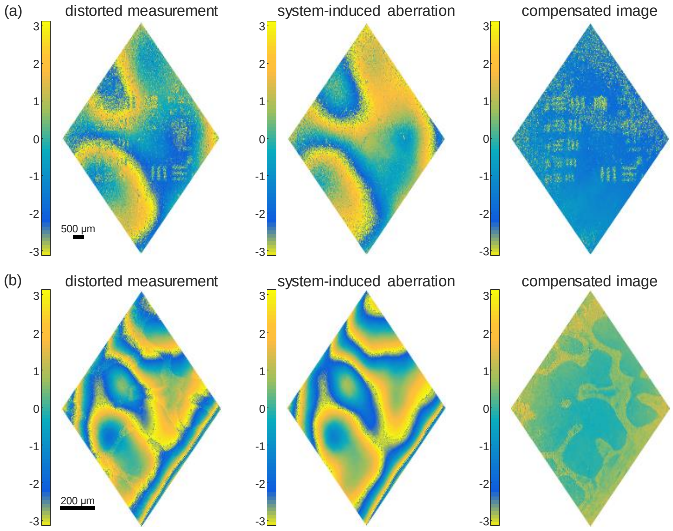

Supplementary Fig. 1 Examples of correcting phase contaminations. (a) Phase images of a standard positive 1951-USAF resolution test target (R3L3S1P, Thorlabs). The original phase image with the sample, the phase image without the sample, and the corrected phase image are shown in order. (b) Corresponding phase images of stained biological tissue from mouse tails. The corresponding scale bars are shown.

Supplementary Figure 1(a) illustrates the original phase image of the sample, the phase image without the sample, and the corrected phase image in order. These images demonstrate that phase contaminations induced from the optical system can be effectively removed. Moreover, similar results for imaging stained biological tissue from mouse tails are displayed in Supplementary Fig. 1(b).

Supplementary Note 3. Operations of the acousto-optic modulator (AOM)

The AOMs we used during experiments were made up of a slender crystal with refractive index sensitive to the sound field and an electro-acoustic transducer. A function generator produced a sinusoidal electronic signal (800 mV), which was then amplified to about 19.2 V. When driven by this amplified sinusoidal electrical signal, a salvo of the sound wave was generated among the crystal. The sound wave dynamically modulated the local optical properties of the crystal, causing a Doppler frequency shift to the light passing through. The shifted frequency equals the frequency of the sound wave.

Supplementary Note 4. Operations of the DMD

As shown in Supplementary Fig. 2, the DMD acts as a two-dimensional blazed grating that reflects the illumination beam in a special diffractive way. These micromirrors would flip at a high refresh rate of 22 kHz with the shinning angles of $+12^{\circ}$ and -12°. In this work, we set the micromirrors corresponding to $+12^{\circ}$ the "on-state", and light reflected by these micromirrors is directed to the sample as the signal beam (the yellow arrow in Supplementary Fig. 2(a)). In contrast, the micromirrors that correspond to -12° mirrors are set as the "off-state", generating the idle beam (the red arrow in Supplementary Fig. 2(a)). As a typical illustration, the upper right inset in Supplementary Fig. 2 shows the structure of 2×2 pixels of the DMD, containing both "on-state" micromirrors and "off-state" micromirrors. Since the axis of rotation is along the diagonal line of these micromirrors, the DMD needs to be rotated 45° to make both the illumination beam and the signal beam horizontal, resulting in a rotated rectangular shape. When observing along the axis of rotation, a conspicuous schematic of the diffracted light from the DMD is illustrated in the lower right inset of Supplementary Fig. 2.

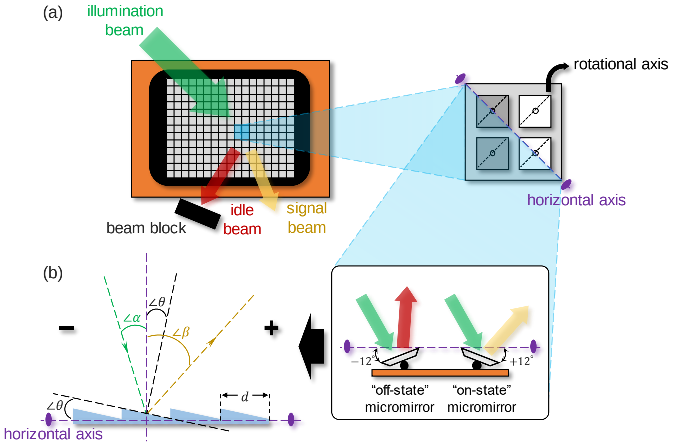

Supplementary Fig. 2 Operational principle of the DMD. (a) A schematic view of the operations of the DMD, in which the illumination beam, idle beam, and the signal beam are denoted by green, red, and yellow, respectively. The inset shows the detailed structure of the micromirrors, showing that the rotation axis is 45° oriented. (b) The illustration of light being diffracted from the DMD, which is simplified as a one-dimensional blazed grating.

For a reflection blazed grating, it is important to maximize the diffraction efficiency for the signal beam, which is the conjunction of single-slit diffraction and multi-slit interference. Mathematically, one-dimensional (1D) angular distribution of the diffracted light can be formulated as an analogy of multi-slit Fraunhofer diffraction, which is listed below:

$$I(\alpha,\beta) = I_0\left(\frac{\sin\xi'}{\xi'}\right)^2\left(\frac{\sin N\delta'/2}{\sin\delta'/2}\right)^2 \qquad \text{(S7)}$$

where α (resp. β) is the incident angle (resp. diffractive angle) for the normal line, N is the number of the slit in one dimension, ξ' is the single-slot diffraction factor of $\frac{\pi a}{\lambda}\left(\sin(\alpha+\theta)-\sin(\beta-\theta)\right)$, and δ' is multi-slit interference factor of $\frac{2\pi d}{\lambda}(\sin\alpha-\sin\beta)$. Here, $\lambda = 532$ nm is the wavelength of the illuminating light and $d = 13.68\mu m \times \sqrt{2} \approx 19.35\mu m$ is the pitch of the DMD. The factor of $\sqrt{2}$ is originated from the layout of the diamond shape so that the effective pitch is the diagonal length. a is the effective length of the single slit, which is slightly smaller than d due to the nonzero shinning angle θ shown in Supplementary Fig. 2(b). For the first coefficient that is determined by the single-slit diffraction, the maximum value can be reached if

$$\alpha+\theta=\beta-\theta \qquad \text{(S8)}$$

For the second coefficient resulted from multi-slit interference, the maximum value can be reached given the following relation can be satisfied:

$$d(\sin\alpha-\sin\beta)=m\lambda \quad m=0,\pm1,\pm2,\cdots \qquad \text{(S9)}$$

For $\theta = 12^{\circ}$, i.e., the "on-state" is chosen as the signal beam, Eqs. S8 and S9 lead to an optimum incident angle $\alpha = 17.92^{\circ}$ and a corresponding diffracted angle $\beta = 41.92^{\circ}$ at $m = 7$. This diffracted angle β will cause the displayed pattern not perpendicular to the propagation direction, inducing a phase ramp across the projected pattern. Nonetheless, the calibration process to correct phase contamination we described above can conveniently ease this affection. Moreover, this configuration also causes compression along the horizontal direction. For example, the length of the diagonal line along the horizontal direction becomes $19.35\ \mu\text{m} \times 768 \times \cos(41.92^{\circ}) \approx 11.1$ mm, while the length of the diagonal line along the vertical direction is not affected. This configuration is also the reason that causes unequal lateral resolution and field of view (FOV) in different directions, leading to a length ratio of 0.74

(horizontal/verticle). Due to this compression, a square pattern displayed by the DMD is transformed into a diamond shape. Notably, if a rectangular pattern is displayed by the DMD, this transformation turns the projected pattern into an asymmetric parallelogram shape. Thus, for visualization purposes, we restricted ourselves to use only square active areas of the DMD throughout this work.

Supplementary Note 5. The ordering of Hadamard bases: square path. For compressive sensing, the ordering of Hadamard bases is crucially important, as information is not evenly distributed among different spatial frequencies for natural scenes. In general, the lower spatial frequency usually contains more information than the higher spatial frequency. As a result, we adopted a square path to transverse Hadamard bases for our reconstruction procedure with inverse fast Hadamard transformation [1], as shown in Supplementary Fig. 3.

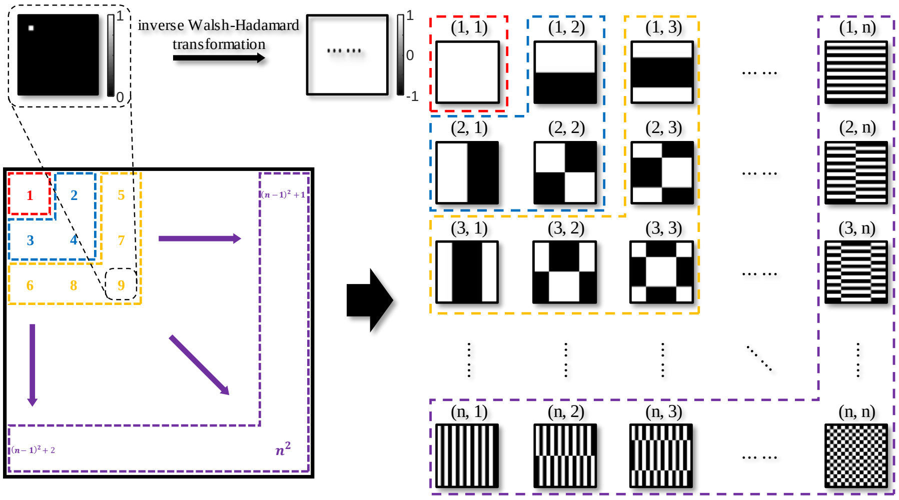

Supplementary Fig. 3 Illustration of the ordering for Hadamard bases. The "square path" was named after the transverse order of the only nonzero component (the white block) in the two-dimensional matrix. The corresponding Hadamard basis was obtained by performing the inverse Walsh-Hadamard transformation to the Dirac-like patterns in the upper left corner.

The name "square path" was originated from the sequence of raster scanning the only nonzero element in the two-dimensional (2D) Dirac-like patterns. As denoted in Supplementary Fig. 3, raster scanning was performed alongside the gradually increased length of the square, i.e., red first, then blue, and followed by yellow. The corresponding ordered Hadamard basis can then be produced by performing a 2D inverse Walsh-Hadamard transformation to the Dirac-like patterns. Because of the particular rule of bitwise operation in Walsh-Hadamard transformation, the number of row m and column n indicate $m - 1$ and $n - 1$ times flipping along each direction with black and white, respectively. For example, the pattern with the white element at $(3,3)$ (shown in the upper left frame in Supplementary Fig. 3), leads to twice flipping along both directions in the generated Hadamard basis. For Hadamard bases within the same reversed "L" shape, they follow an increasing order of $m + n$, which is numbered in Supplementary Fig. 3.

Supplementary Note 6. Investigations on compressive sensing under different noise levels

In practice, measurement noises contributed from shot noises of light and electronic noises of equipment, causing degradation in image quality. Using numerical tools, here, we quantify how this effect impacts the performance of compressive sensing with different sampling ratios (SRs).

The original holographic image S was set with a resolution of 256 × 256. We adopted a direct inverse basis transformation with a square path for compressive sensing, as described in main text. For simplicity, measurement noises were simulated as white Gaussian noises with various standard deviations [1]. In particular, noise levels ranging from 0 to 1% of the averaged measurement values were considered. For compressive sensing, various SRs at 3.125%, 6.25%, 12.5%, 25%, 50%, and 100% were employed to reconstruct images O_{CS} under different noise levels. A first-order correlation function between the reconstructed and original images, defined as $|\langle O_{CS}, S\rangle| / (|\langle O_{CS}, O_{CS}\rangle||\langle S, S\rangle|)^{1/2}$, was used to quantify the similarities between these two holographic images.

Supplementary Figure 4(a) plots correlations as a function of noise levels when holographic images were reconstructed at different SRs. For all cases, the correlations decrease as the noises become large. However, correlations correspond to different SRs decay at different speeds. In general, the larger the SR is, the faster the correlation decays. Thus, although the reconstruction process with a small

SR performs worse for small measurement noises, it turns out to be better when measurement noises become large. This observation indicates that compressive sensing with a smaller SR, which concentrates more on the lower spatial frequency, is more robust to measurement noises. In our experiment, the noise level is around 0.1% of the measurement, denoted as a yellow area in the figure. To identify which SR is the most suitable one under the current experimental condition, we then fixed the noise level of 0.1% in the following simulation. Under such conditions, blue circles in Supplementary Fig. 4(b) describe the decreased correlation solely induced by the noise, while red circles describe the evolution of the correlation purely resulted from the changing of SRs in compressive sensing. Notably, the trends of these two effects indicate that there exists a suitable SR under the current noise level. By multiplying these two effects, yellow circles represent the combined effect. One could see that the maximum correlation is achieved at SR = 25%. This result explains why the reconstructed images with SR = 100% and 50% look worse than those with SR = 25% and 12.5% in main text. Nonetheless, we emphasize here that although a large SR is susceptible to measurement noises, it still provides a high-resolution image, as the decreased correlation is mainly contributed from the noisy background but not from the fine feature.

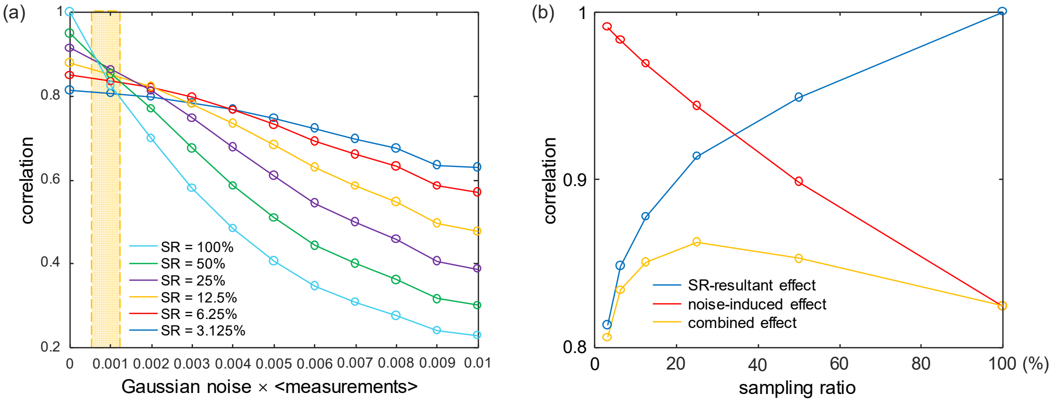

Supplementary Fig. 4 Simulation results on compressive sensing under different noise levels. (a) Correlations between the reconstructed and original images as a function of different noise levels. Compressive sensing realized with different sampling ratios (SRs) were investigated, which are labeled with different colors. The yellow area represents the actual noise levels during experiments. (b) After fixing the noise level as 0.1% of the measurement value, the SR-resultant effect and the noise-induced effect are represented using blue and red circles as a function of the SR. The combined effect is represented using yellow circles, exhibiting a maximum value at SR = 25%.

Supplementary Note 7. Holographic results of using the strategy of 2 × 2 pixels binning

As we mentioned in main text, the scalability of our system can also be reflected in choosing a different binning strategy. Here, we showed the holographic results of using the strategy of 2 × 2 pixels binning. For this strategy, 256 × 256 superpixels at the center part of the DMD were employed to demonstrate the imaging performance. The system was also operated under large-FOV mode with a 1:1 imaging relation of the 4*f* system, expect a FOV of 9.91 mm × 7.37 mm and a resolution of 38.7 μm × 28.8 μm. The corresponding raw data are available in public repository Zenodo [2]. Thus, element 1 of group 4 (31.25-μm width) in the resolution target is the best metric to be identified.

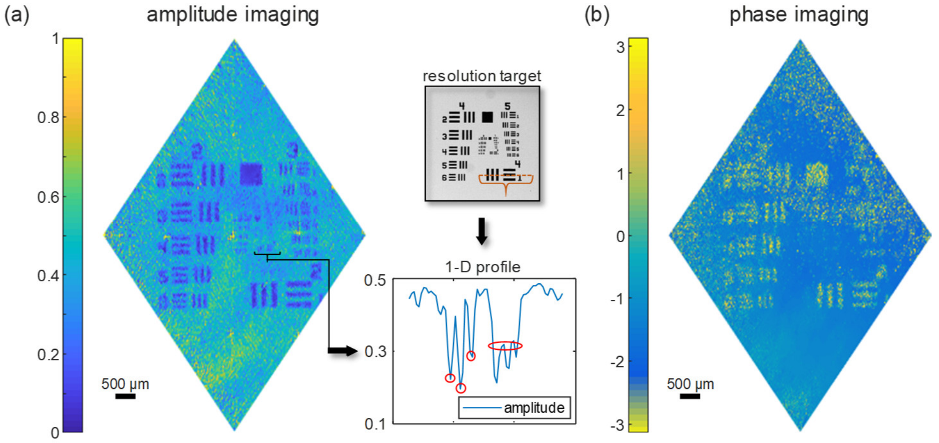

Supplementary Fig. 5 Holographic results of using the strategy of 2 × 2 pixels binning. (a) The amplitude image of the resolution target. The upper inset shows the amplitude image captured by conventional microscopy. The 1D profile in the lower inset shows that the system can resolve element 1 of group 4 (31.25-μm width). (b) the phase image of the resolution target. The corresponding scale bar is 500 μm.

Supplementary Figures 5(a) and (b) show the reconstructed holographic images for groups 2-4. For comparison, the image captured by the conventional microscope (with a much smaller FOV but finer resolution) is also displayed in the upper inset. For visualization purposes, 1D profile signified by the black bracket in the amplitude imaging is plotted in the inset below, exhibiting three well-separated narrow dips (vertical lines) and one wide dip (horizontal line). These results suggest that the resolution of the built holographic system meets the theoretical expectation, confirming the scalability of this holographic system.

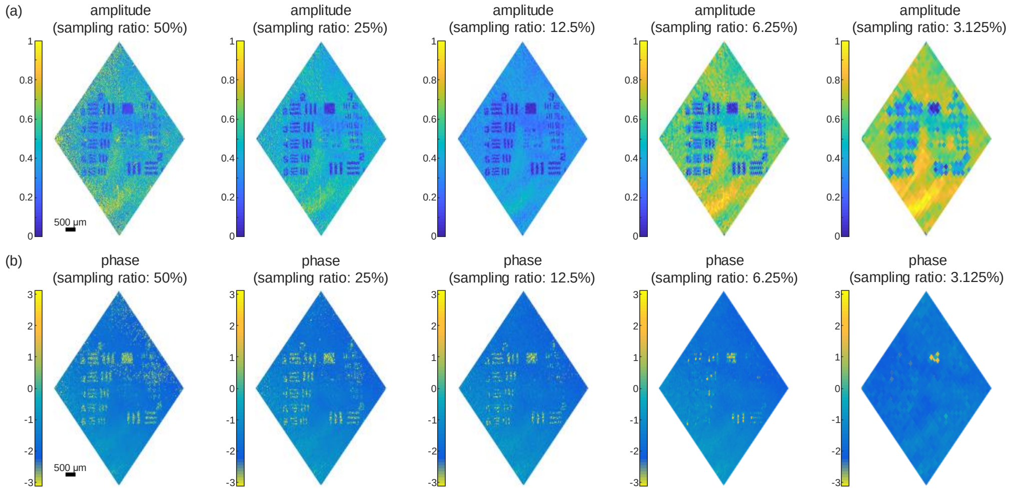

Supplementary Fig. 6 Holographic results of imaging the resolution target with compressive sensing. (a)(b) The amplitude and phase images reconstructed with different sampling ratios of 50%, 25%, 12.5%, 6.25%, 3.125%. The corresponding scale bar is 500 μm.

Similar to the procedures done in main text, we also showed the corresponding images reconstructed with compressive sensing in Supplementary Fig. 6. Again, these results show that compressive sensing with SRs down to12.5% can produce acceptable results.

Supplementary Note 8. Holographic results of a quantitative phase resolution target

To quantify the imaging capability in phase, a quantitative phase resolution target (QPT, BenchMark Tec) was imaged. This type of phase target is fabricated by coating transparent materials on a piece of glass. Specifically, phase patterns with the same size as groups 6-7 of the USAF standard resolution target are provided, allowing us to gauge the resolution in phase. While operating under the high-resolution mode, Supplementary Fig. 7 shows the reconstructed amplitude and phase images of this phase target. Due to the low contrast in transmission, the amplitude image shown in Supplementary Fig. 7(a) is vague. Nevertheless, the phase image shown in Supplementary Fig. 7(b) exhibits great performance, manifesting delicate phase patterns that are almost blind in the amplitude counterpart. The corresponding raw data are available in public repository Zenodo [2]. Specifically, we also quantified that the smallest structure that can be distinguished in phase is element 6 of group 6 (4.386-μm width), with a corresponding 1D profile displayed in the upper inset. This result agrees with the theoretical resolution under the high-resolution mode, which is 5.80 μm × 4.31 μm.

Next, appraising whether the reconstructed phase value is quantitatively correct is another important issue. Given a coating thickness of 250 nm and a refractive index of 1.52, the phase difference between the phase pattern and the background is estimated to be $\Delta\varphi \approx 1.795$ rads for the green light. Here, we examined the bar located at the bottom of the phase target, enclosed in a dashed rectangular. To minimize statistical errors, the same area was imaged four times. 1D profile that crosses both the bar and the background is depicted in the lower inset using blue circles, exhibiting a stepped structure. The errorbars represent the standard deviation of four independent measurements, resulting in an averaged fluctuation of about 0.137 rads. The averaged phase difference was estimated to be $\Delta\varphi_{\mathrm{exp}} \approx 1.691$ rads, giving a phase error of only 0.104 rads ($\leq \lambda/60$). These results confirm that the developed SPH is quantitatively accurate to retrieve phase, showing prospects in biophotonics.

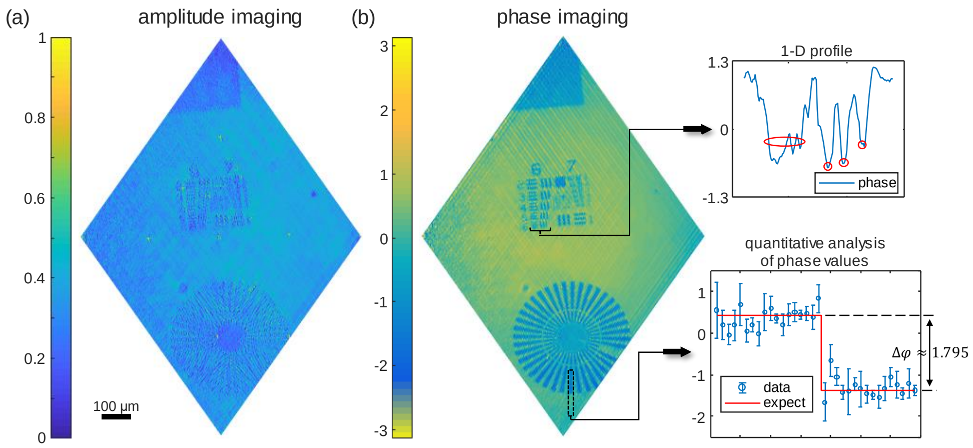

Supplementary Fig. 7 Holographic results of imaging a quantitative phase resolution target under high-resolution mode. (a) The amplitude image of the quantitative phase resolution target, showing poor contrast. (b) The phase image of the quantitative phase resolution target, showing clear phase patterns. Upper inset: the corresponding one-dimensional profile of element 6 of group 6 (4.386-μm width). Lower inset: quantitative analysis on the accuracy of the reconstructed phase values (actual phase difference $\Delta\varphi = 1.795$ rads). The data are presented as mean values and standard deviations, resulting in a phase error of about 0.104 rads (from $n = 4$ independent measurements). The corresponding scale bar is 100 μm.

Moreover, Supplementary Fig. 8 shows the holographic results with compressive sensing. Similarly, when the SR decreases, the detailed structure gradually becomes obscure. Nonetheless, the holographic result reconstructed when SR = 12.5% is still acceptable.

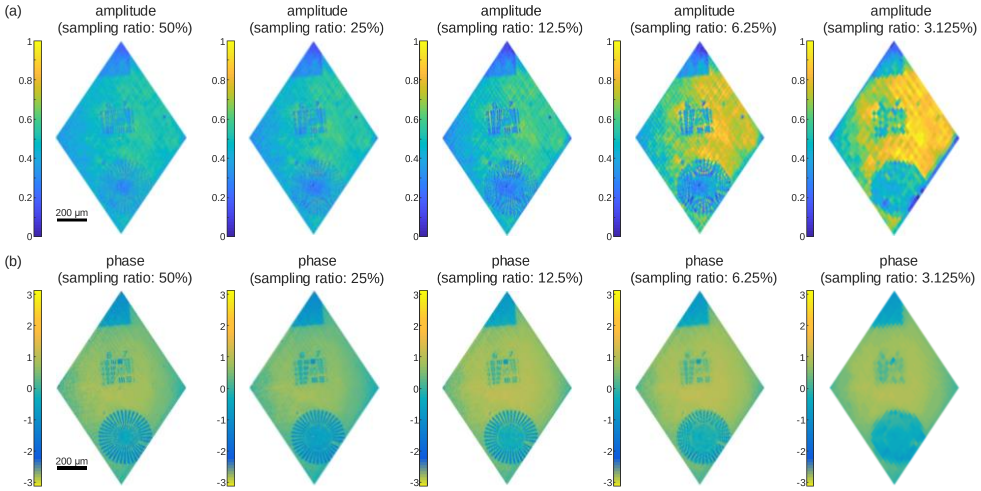

Supplementary Fig. 8 Holographic results of imaging the phase resolution target with compressive sensing. (a)(b) The amplitude and phase images reconstructed with different sampling ratios of 50%, 25%, 12.5%, 6.25%, and 3.125%. The corresponding scale bar is 200 μm.

Supplementary Note 9. Additional holographic images of stained tissue from mouse tails

To further demonstrate the imaging capability for stained biological tissue, we imaged another piece of slice from mouse tails, which is 10-μm thick. Supplementary Figure 9 shows a bright-field image of this slice, captured using a conventional microscope. In this image, several types of tissue such as muscle, cortical bone, and cancellous bone are visually identified. Supplementary Figures 9(b), (c), and (d) show a series of reconstructed holographic images for different parts of mouse tail. The corresponding raw data are available in public repository Zenodo [2]. Again, for this stained slice, the amplitude images are in good agreement with the one shown in Supplementary Fig. 9(a), manifesting great distinctions among different types of tissue. Like the one presented in Supplementary Fig. 3 of main text, the reconstructed phase images are analogous to their amplitude counterparts.

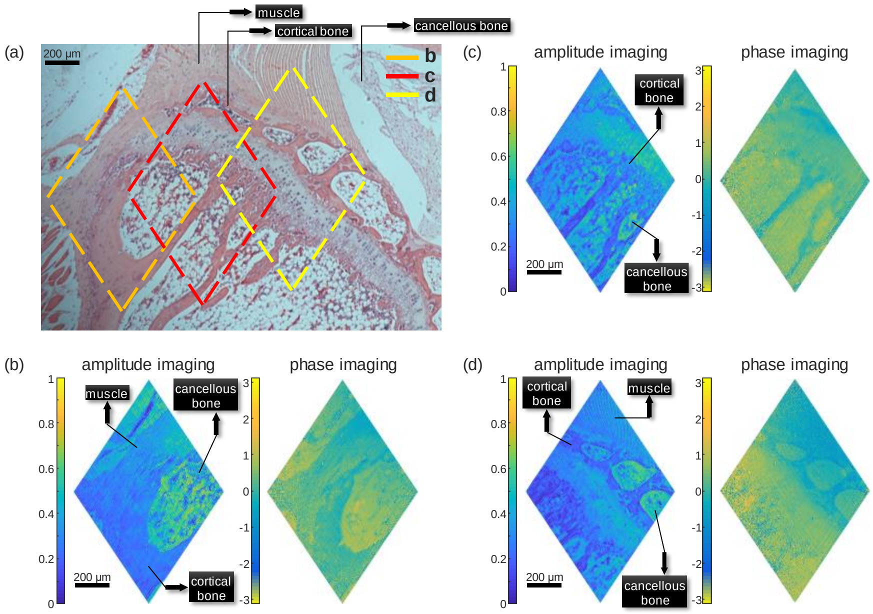

Supplementary Fig. 9 Holographic performance of stained mouse tail tissue with 10-μm thickness. (a) The image of a slice of a 10-μm-thick stained tissue from mouse tails, captured using a conventional microscope. Three diamond-shaped boxes represent the area being imaged by the holographic system. (b)-(d) The reconstructed amplitude and phase images for different parts of the stained tissue. The corresponding scale bar is 200 μm.

Supplementary Note 10. Additional holographic images of unstained tissue from mouse brains

To show the imaging capability for unstained tissue, we also imaged a 100-μm-thick slice of unstained mouse brain. Supplementary Figure 10(a) shows a bright-field image of this slice, captured using a conventional microscope. The imaged part of the tissue contains white matter and grey matter. Since the unstained slice is relatively thick with roughly the same transmission across the imaging region, it is challenging for the conventional microscope to provide good contrast. Supplementary Figures 10(b), (c), and (d) show a series of reconstructed holographic images for different parts of the mouse brain, denoted by three labeled diamond-shaped boxes in Supplementary Fig. 10(a). The corresponding raw data are available in public repository Zenodo [2]. As expected, the features in amplitude images match well with that at corresponding areas in Supplementary Fig. 10(a), which do not provide too much information with good contrast. As a comparison, phase images provide much better contrast, revealing many detailed structures that are indiscernible in their amplitude counterparts.

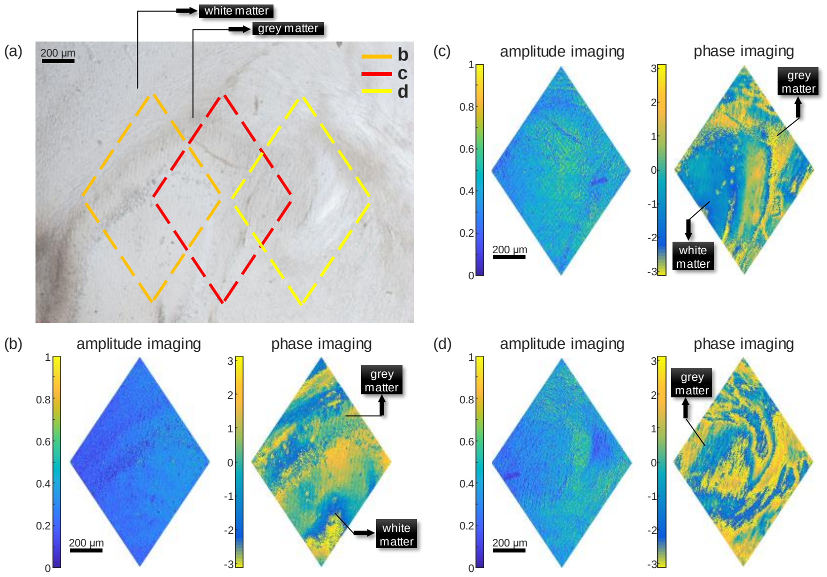

Supplementary Fig. 10 Holographic performance of unstained mouse brain tissue with 100-μm thickness. (a) The image of a slice of 100-μm-thick unstained tissue from mouse brains, captured using a conventional microscope. Three diamond-shaped boxes represent the area being imaged by the holographic system. (b)-(d) The reconstructed amplitude and phase images for different parts of the unstained tissue. The corresponding scale bar is 200 μm.

We also imaged an even thicker slice of unstained mouse brain with a thickness of 120 μm. Supplementary Figure 11(a) shows a bright-field image of this slice, captured using a conventional microscope. The imaged part contains the ventral part of the lateral septal nucleus, inferior colliculus, and white matter. Supplementary Figures 11(b), (c), and (d) show a series of reconstructed holographic images for different parts of mouse brain, denoted by three labeled diamond-shaped boxes in Supplementary Fig. 11(a). Again, phase images provide much better contrast, compared to their amplitude counterparts.

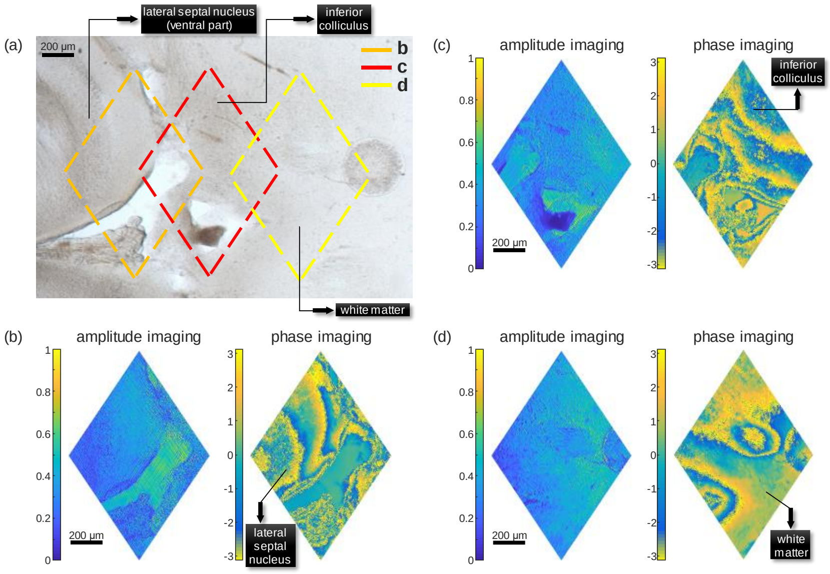

Supplementary Fig. 11 Holographic performance of unstained mouse brain tissue with 120-μm thickness. (a) The image of a slice of 120-μm-thick unstained tissue from mouse brains, captured using a conventional microscope. Three diamond-shaped boxes represent the area being imaged by the holographic system. (b)-(d) The reconstructed amplitude and phase images for different parts of the stained tissue. The corresponding scale bar is 200 μm.

Very thin unstained tissue from mouse brains down to 10-μm thick was also imaged using our holographic system. Supplementary Figure 12(a) shows a bright-field image of this slice, captured using a conventional microscope. The imaged part contains white matter and grey matter. Supplementary Figures 12(b), (c), and (d) show a series of reconstructed holographic images for different parts of mouse brain, denoted by three labeled diamond-shaped boxes in Supplementary Fig. 12(a). For such a thin tissue, it is hard to see details through the amplitude images. Nonetheless, phase images still provide rich information with good contrast.

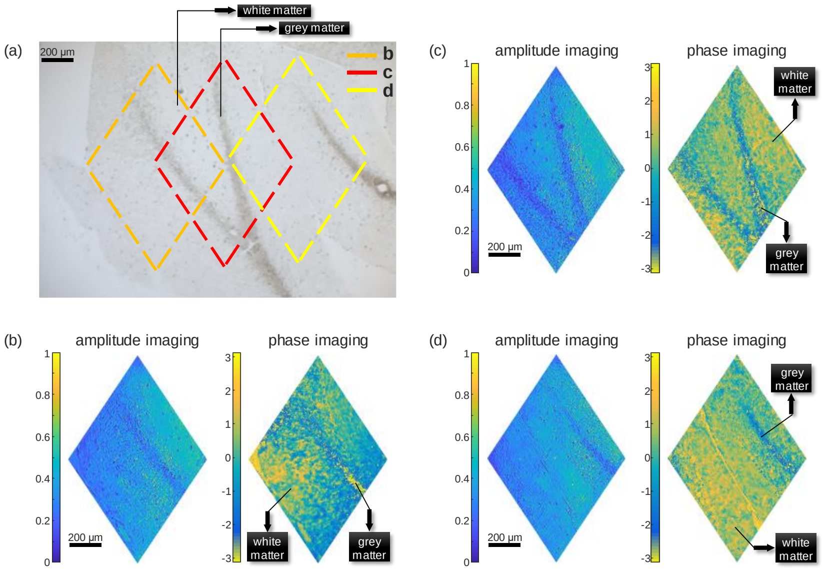

Supplementary Fig. 12 Holographic performance of unstained mouse brain tissue with 10-μm thickness. (a) The image of a slice of 10-μm-thick unstained tissue from mouse brains, captured using a conventional microscope. Three diamond-shaped boxes represent the area being imaged by the holographic system. (b)-(d) The reconstructed amplitude and phase images for different parts of the stained tissue. The corresponding scale bar is 200 μm.

Supplementary Note 11. Parameters in the large-FOV mode and the high-resolution mode

During experiments, we employed a square area with 768 × 768 effective pixels of the DMD. Since the pitch of the DMD is 19.35 μm (calculated above), the length of the diagonal lines along both the horizontal and vertical directions for the generated pattern are the same to be 19.35 μm × 768 ≈ 14.9 mm.

For the large-FOV mode demonstrated in this work, the two lenses employed for the $4f$ system are the same (AC254-125-A, Thorlabs). Thus, the surface of the DMD is 1:1 imaged to the surface of the sample. Considering a diffraction angle $\beta = 41.92^{\circ}$ that causes compression along the horizontal direction, the lengths of the diagonal lines along the horizontal and vertical directions are 14.9 mm and 14.9 mm × cos(41.92°) ≈ 11.1 mm, respectively. Since a 3 × 3 pixels binning was employed, the corresponding resolution along the horizontal and vertical directions are 19.35 μm × 3 ≈ 58.0 μm and 19.35 μm × 3 × cos(41.92°) ≈ 43.1 μm, respectively. A larger FOV can be further achieved by amplifying the projected patterns using a different $4f$ system.

For the high-resolution mode demonstrated in this work, the two lenses employed for the $4f$ system have focal lengths of 300 mm (AC254-300-A, Thorlabs) and 30 mm (AC254-30-A, Thorlabs), respectively. Thus, the surface of the DMD is demagnified by a factor of 10 when imaged to the surface of the sample. In this case, both the FOV and the resolution are scaled down by a factor of 10 compared to that in the large-FOV mode. Thus, the FOV and the resolution along the horizontal and vertical directions are 1.49 mm × 1.11 mm and 5.80 μm × 4.31 μm, respectively." A finer resolution can be further achieved by using a $4f$ system with a larger minification. However, such a $4f$ system requires special care due to the emergence of optical aberrations.

References

1. Z. Zhang, X. Wang, G. Zheng, and J. Zhong, "Hadamard single-pixel imaging versus Fourier single-pixel imaging," Optics Express **25**, 19619-19639 (2017).

2. D. Wu, J. Luo, G. Huang, Y. Feng, X. Feng, R. Zhang, Y. Shen, and Z. Li, "Source Data for "Imaging biological tissue with high-throughput single-pixel compressive holography"," Zenodo. http://doi.org/10.5281/zenodo.5105874 (2021).
