# Supplementary material for: Imaging biological tissue with high-throughput single-pixel compressive holography
Source: Nat Commun. 2021 Aug 5;12:4712. doi: 10.1038/s41467-021-24990-0 (PMC8342474; doi:10.1038/s41467-021-24990-0)
Supplement: Supplementary file 2 — Reporting Summary [file 41467_2021_24990_MOESM2_ESM.pdf]

nature research

Corresponding author(s): Yuecheng Shen and Zhaohui Li

Last updated by author(s): Jul 8, 2021

Reporting Summary

Nature Research wishes to improve the reproducibility of the work that we publish. This form provides structure for consistency and transparency in reporting. For further information on Nature Research policies, see our Editorial Policies and the Editorial Policy Checklist.

Statistics

For all statistical analyses, confirm that the following items are present in the figure legend, table legend, main text, or Methods section.

n/a	Confirmed	
☐	☒	The exact sample size (*n*) for each experimental group/condition, given as a discrete number and unit of measurement
☐	☒	A statement on whether measurements were taken from distinct samples or whether the same sample was measured repeatedly
☒	☐	The statistical test(s) used AND whether they are one- or two-sided<br>*Only common tests should be described solely by name; describe more complex techniques in the Methods section.*
☒	☐	A description of all covariates tested
☒	☐	A description of any assumptions or corrections, such as tests of normality and adjustment for multiple comparisons
☐	☒	A full description of the statistical parameters including central tendency (e.g. means) or other basic estimates (e.g. regression coefficient) AND variation (e.g. standard deviation) or associated estimates of uncertainty (e.g. confidence intervals)
☒	☐	For null hypothesis testing, the test statistic (e.g. *F*, *t*, *r*) with confidence intervals, effect sizes, degrees of freedom and *P* value noted<br>*Give P values as exact values whenever suitable.*
☒	☐	For Bayesian analysis, information on the choice of priors and Markov chain Monte Carlo settings
☒	☐	For hierarchical and complex designs, identification of the appropriate level for tests and full reporting of outcomes
☒	☐	Estimates of effect sizes (e.g. Cohen's *d*, Pearson's *r*), indicating how they were calculated

Our web collection on statistics for biologists contains articles on many of the points above.

Software and code

Policy information about availability of computer code

Data collection	Data collection is accomplished by executing the files in the folder named "data acquisition", which can be accessed in the republic repository Zenodo.<br>The DOI link listed below:<br>Daixuan Wu. (2021). Source Data for "Imaging biological tissue with high-throughput single-pixel compressive holography" [Data set]. Zenodo. http://doi.org/10.5281/zenodo.5089852<br>An integrated C++ source file named 'joint_256bitplane8.cpp' controls the digital micromirror device (DMD) to display the desired Hadamard-like patterns with full speed and the data acquiescing card (DAC) to acquire data. We tested these files with Visual Studio 2019 (community version). This folder also includes all necessary files of Visual Studio C++ project to control both the DMD and the DAC.
Data analysis	The experimentally measured raw data are analyzed using the computer program MATLAB (2016b version and above). The MATLAB codes are showcased for holographic reconstruction and compressive sensing in the folder named "holographic reconstruction" as practical demonstration. The code is also accessed in the republic repository Zenodo, which is included in 'code availability' section.<br>The DOI link listed below:<br>Daixuan Wu. (2021, July 6). Supplementary Code for "Imaging biological tissue with high-throughput single-pixel compressive holography". Zenodo. http://doi.org/10.5281/zenodo.5089869<br>*) singlepixel_dataprocessing_complex.m : This .m file is placed in both subfolders of "stained tissue from mouse tails" and "unstained tissue from mouse brains" so that it aims at reading the measured raw data and calls the supporting subfunctions to reconstruct holographic images with compressive sensing.

For manuscripts utilizing custom algorithms or software that are central to the research but not yet described in published literature, software must be made available to editors and reviewers. We strongly encourage code deposition in a community repository (e.g. GitHub). See the Nature Research guidelines for submitting code & software for further information.

Data

Policy information about availability of data

All manuscripts must include a data availability statement. This statement should provide the following information, where applicable:

- Accession codes, unique identifiers, or web links for publicly available datasets
- A list of figures that have associated raw data
- A description of any restrictions on data availability

Data to produce holographic images in manuscript have been deposited in public repository Zenodo. Additional data to produce holographic images in Supplementary Information are available from the corresponding authors upon request, as they occupy a lot of spaces but have similar structures.

Field-specific reporting

Please select the one below that is the best fit for your research. If you are not sure, read the appropriate sections before making your selection.

[x] Life sciences [ ] Behavioural & social sciences [ ] Ecological, evolutionary & environmental sciences

For a reference copy of the document with all sections, see nature.com/documents/nr-reporting-summary-flat.pdf

Life sciences study design

All studies must disclose on these points even when the disclosure is negative.

Sample size	Since we aim at proving the holographic imaging capability of SPH system, a number of biological samples (two slices of stained tissue from mouse tails and four slices of unstained tissue from mouse brains) were used for imaging purposes. Although no sample size calculation was performed, stained tissues and unstained tissues with different thicknesses were sufficient for us to verify the imaging capability.
Data exclusions	No data was excluded from the analyses.
Replication	To help the readers to replicate holographic images for biological sample, the measured raw data, converted MATLAB data were provided as source data available in the public repository Zenodo, and the supporting codes for data collecting and reconstructing holographic images were also provided in the public repository Zenodo. All materials were enclosed in republic repository alongside with the instruction. We confirm that the holographic imaging of stained tissue from mouse tails and unstained tissue from mouse brains can be replicated successfully.
Randomization	For stained tissue from mouse tails, we prepared 8 slices with 10-µm thickness as a group, which was defined as 'group of stained tissue'. For unstained tissue from mouse brains, we divided samples with [10, 80, 100, 120]-µm thickness into four groups and each group had 3 samples, which was defined as 'group of unstained tissue with [10, 80, 100, 120]-µm thickness'. Holographic images for slices of biological tissue can be reconstructed successfully using the developed high-throughput single-pixel holography. Therefore, several slices, i.e., two slices of stained tissue from mouse tails (randomly chosen from 'group of stained tissue') and four slices of unstained tissue from mouse brains (each slice was randomly chosen from 'group of unstained tissue with [10, 80, 100, 120]-µm thickness'), were deposited as targets to be imaged. We declare that there is no predetermination of samples during our experiments.
Blinding	The investigators were blinded to group allocation during data collection and analysis.

Reporting for specific materials, systems and methods

We require information from authors about some types of materials, experimental systems and methods used in many studies. Here, indicate whether each material, system or method listed is relevant to your study. If you are not sure if a list item applies to your research, read the appropriate section before selecting a response.

Materials & experimental systems

n/a	Involved in the study
[x]	[ ] Antibodies
[x]	[ ] Eukaryotic cell lines
[x]	[ ] Palaeontology and archaeology
[ ]	[x] Animals and other organisms
[x]	[ ] Human research participants
[x]	[ ] Clinical data
[x]	[ ] Dual use research of concern

Methods

n/a	Involved in the study
[x]	[ ] ChIP-seq
[x]	[ ] Flow cytometry
[x]	[ ] MRI-based neuroimaging

Animals and other organisms

Policy information about studies involving animals; ARRIVE guidelines recommended for reporting animal research

Laboratory animals	biological samples (slices of stained tissue from mouse tails and slices of unstained tissue from mouse brains) used for holographic imaging in this study were harvested from two- to three-month-old female mice (C57BL/6N-Hsd: Athymic Nude-FoxlNU, Harlan). These mice were raised in mouse cages with standard disinfection. We kept on a reversed 12:12 light/dark cycle (lights on from 6 a.m. to 6 p.m.). The ambient temperature was kept in the range of 18-22 degrees Celsius, as well as the humility was in the range of 50-60 %.
Wild animals	The study did not involve wild animals.
Field-collected samples	The study did not involve samples collected from the field.
Ethics oversight	We affirmed that all procedures were carried out in conformity with ethical regulations approved by the Institutional Animal Care and Use Committee, Sun Yat-sen University.

Note that full information on the approval of the study protocol must also be provided in the manuscript.
